# A Suggested Approach for Management of Pediatric Asthma During the COVID-19 Pandemic

**DOI:** 10.3389/fped.2020.563093

**Published:** 2020-09-25

**Authors:** Bo Ding, Yanming Lu

**Affiliations:** Department of Pediatrics, South Hospital of Renji Hospital, Shanghai Jiaotong University School of Medicine, Shanghai, China

**Keywords:** asthma, COVID-19, management, pediatric, pandemic (COVID-19)

## Abstract

Asthma is a prevalent pediatric disease causing important health, economic, and emotional burdens around the world. Asthma attacks can be controlled with standardized management, but no cure exists for the disease. Many attacks are triggered by respiratory tract infections and children with basic airway diseases are at high risk for developing severe or critical illnesses. The new COVID-19 pandemic threatens to disrupt children's asthma control management and we have set out to summarize the main factors that need to be considered by pediatricians treating children with asthma at times like these. We discuss the intrinsic nature of asthma and its treatment, and the effects of irregular treatment giving recommendations such as the use of the WeChat platform and WeChat Official Accounts for follow-ups to improve children's asthma compliance during the pandemic. We also cover the COVID-19 protection strategies, and the importance of stress reduction, a balanced diet, exercise, and the avoidance of known attack triggers for maintaining good control of asthma during the pandemic.

## Background Information

Asthma is the most common chronic respiratory disease in children. Approximately 300 million people in the world (4.3%) have asthma, and 14% of children suffer from the disease (1, 2). The total prevalence of asthma has increased, but it differs in various countries and cities (3). The prevalence of childhood asthma in China increased from 1.97% in 2000 to 3.02% in 2010, due to the rapid economic development, accelerated urbanization, and environmental exposure to pollutants (4, 5). Moreover, the prevalence in Shanghai alone increased from 4.52 to 7.57% (6, 7). China has a large population, and a slight increase in the prevalence results in a significant increase in the number of patients. Asthma is a challenging health problem, and the number of patients worldwide is predicted to increase by 100 million in the next 10 years (2). Asthma also creates an economic burden on families, and diminishes the quality of life of poor children, causes emotional disorders, and may lead to repeated absences from school and other problems (3). The control of children's asthma in Asia is not ideal, and the complete control rate stands at only 6.1–67.1% of patients (8). A 2006 survey of children with asthma from 0–16 years in the Asia-Pacific region showed that 53.4% (528/988) of them had uncontrolled disease (9). Liu et al. (10) surveyed 497 cases of asthma in children 0–14 years and found an uncontrolled rate of 34.3%. Lack of standardized asthma control strategies and treatment is the leading cause of the high uncontrolled cases rate (11). The main factors affecting the severity of asthma attacks include children and guardians' insufficient understanding of the disease, insufficient treatment, poor medication adherence, exposure to poor environments (such as dusty rooms or tobacco smoke), and irregular follow-up visits after diagnosis (9–11). Respiratory tract infections are the most common triggers of asthma in children (87.9%). Other triggering factors include exercise, specific food consumption, exposure to house dust, and mood changes (3). Standardized management has been proven beneficial to control asthma attacks and improves the prognosis for the children. The asthma control rate can reach 81.8% with strict annual follow ups, and 83.2% with strict follow ups for 2 years (8).

## Covid-19 and Its Effect on Asthma

In December 2019, patients with pneumonia caused by a new type of Coronavirus were identified in Wuhan City (Hubei Province) in China. With the spread of the epidemic, Coronavirus infections in children have appeared in other regions of China and abroad. The World Health Organization named the virus severe acute respiratory syndrome -Coronavirus-2 (SARS-CoV2) (12). SARS-CoV2 are β-coronaviruses. Coronaviruses are a subfamily of orthocoronavirinae in the family of coronaviridae, which can be divided into 4 genera: α, β, γ, and δ. Alpha and beta-coronaviruses only infect mammals, while gamma and delta-coronaviruses mainly infect birds (although a few of those can also infect mammals). Coronaviruses that can infect humans include 229E and NL63 in the alpha genus, OC43 and HPU in the beta genus, Middle East Respiratory Syndrome-related coronavirus (MERSr-CoV), Severe Acute Respiratory Syndrome-related coronavirus (SARSr-CoV), and the 2019 novel coronavirus (SARS-CoV2) that causes the disease COVID-19 (13). Coronaviruses are responsible for a variety of diseases in humans and animals, and can cause respiratory, digestive, and nervous system disease. Disease in humans is mainly related to a variety of respiratory syndromes including the common cold, bronchitis, and pneumonia (13, 14).

Most acute childhood asthma attacks are associated with acute viral respiratory infections (11). Globally, 10–30% of upper respiratory tract infections are caused by coronaviruses, and these occupy the second place among viruses that cause common colds (14). Children with asthma have an imbalance of TH1/TH2 cells and altered concentrations of serum cytokines. Long-term use of inhaled glucocorticoids can reduce interleukin-2 content, weaken the T-cell proliferation and phagocytosis abilities. These changes increase the risk of microorganism infections such as viral infections (15, 16). It is also known that patients with asthma have a greater risk of serious outcomes with common cold viral infections compared to patients without asthma (15). However, evidence from the recent COVID-19 pandemic has failed to identify asthma as a significant risk factor for development of COVID-19 (15). Zhang et al. (17) in a case series of 140 patients with community acquired COVID-19 from Wuhan, China did not find any self-reported allergies like asthma, allergic rhinitis, and atopic dermatitis in any of their patients. In another large study, Du et al. (18) have reported that children with allergic diseases like asthma, allergic rhinitis do not have a higher incidence of COVID-19. They also reported no difference in the clinical features, laboratory, and immunological findings of COVID-19 between allergic and non-allergic patients.

Nevertheless, in order to prevent and control the COVID-19 disease, people need to self-isolate at home, and such life style changes may affect children's asthma control: (1) reduced cross-infection rates may result in reduced asthma exacerbations, and the patients guardians may discontinue asthma treatments; (2) doctor follow-ups may be avoided for the fear of SARS-CoV2 transmission; (3) increased exposure to indoor allergens; (4) parental anxiety may affect children; (5) lack of exercise and uncontrolled diet may lead to excessive weight gain; (6) parents of children with asthma need to focus on coronavirus prevention strategies. These considerations are important for pediatricians to manage children with asthma during the COVID-19 pandemic.

## Strict Adherence to Standard Asthma Treatments Should Continue During the Pandemic

### The Intrinsic Nature and Treatment of Asthma

Asthma can start at any age, but mostly starts before school age due to genetic susceptibility and incidence of viral infections (5, 19). National epidemiological data in 2010 showed that asthma occurred in 36.8% of infants, 37.5% of preschoolers, and 25.7% of school-aged children (5). Allergic inflammation is thought to persist in the lower respiratory tract mucosa in patients with asthma, even during remission periods. However, the severity of the lower respiratory tract inflammation can be alleviated, with reduction of swelling in the respiratory tract mucosa, reduction of glandular secretion in the lower respiratory tract, weakening of smooth muscle spasms, and others (20). Asthma attacks recur after encounters with susceptible factors. Typical asthma can be triggered by many factors, and has high recurrence rates, but is typically seasonal. Therefore, children with asthma need long-term treatment and regular follow-ups. Dynamic and personalized adjustments to the treatment plan are usually needed.

Inhaled corticosteroids (ICSs) are an indispensable part of the treatment of childhood asthma. Both domestic and international guidelines recommend ICSs as the top choice for mild and chronic asthma treatment (21, 22). ICSs inhibit the expression and synthesis of multiple cytokines, inhibit the release of inflammatory mediators by inflammatory cells, and block the occurrence and development of allergic inflammation in the respiratory tract through multiple pathways. ICSs can allow repair of inflammation-related damage of respiratory tract tissues, they reduce edema, congestion, and secretions in the respiratory tract mucosa, and relieve bronchial smooth muscle tension (20). Compared with systemic corticosteroid, ICSs get absorbed directly by the respiratory tract tissues with the following advantages: (1) ICSs are administered through the same route allergens get inhaled, and act directly on the airway mucosa with strong localized treatment effects; (2) they avoid gastrointestinal absorption, and require one tenth of the systemic dosage, avoiding or reducing potential side effects caused by systemic corticosteroid usage; (3) they simply medical procedures, reduce medical costs, and increase patient compliance (20, 23). However, only 58.7% of patients choose to use ICSs (7). Many parents fear the potential adverse side effect of corticosteroids, and this has become an independent risk factor for acute asthma attacks and for the development of chronic airflow limitation (3). If the medication is insufficient and the treatment is not standardized, even if the child does not have an acute asthma attack during the COVID-19 pandemic, an asthma attack may appear after resuming school due to increased exposure to respiratory infections, environmental exposures, and stress; that is “Back to School Asthma (BTS)” may ensue. In the Northern hemisphere, BTS accounts for 20–25% of asthma exacerbations requiring hospitalization (24). The impact of long-term ICS use on children's height continues to be of concern to parents and doctors. A controlled study conducted abroad in 5–13 year-old children with mild to moderate asthma showed that after 400 μg of budesonide daily for an average of 5 consecutive years, the final mean height of the experimental group was 1.2 cm smaller than that of the control group (25). On the other hand, poor asthma control can affect children's rest; the use of frequent oral medication can affect children's appetite, and in the long run, it can also reduce the height of children who do not use ICS treatment (22).

### Irregular Treatment Results Affect Lung Function and Increase the Risk for COPD

The goal of asthma treatment should be to completely cure it. However, the current medical conditions do not allow for that to happen (20). Of all life stages, childhood poses the greatest risk for asthma development. Studies have shown that children with asthma and impaired lung function are less likely to experience remission at the age of 7 than their younger age counterparts, and the age at which asthma begins is inversely associated with the rate of asthma remissions (the remission rate of asthma in children is 3.7 times that of adults, and some children with asthma develop a natural remission) (26, 27). Asthma remission is generally defined as the absence of respiratory symptoms without use of anti-asthma medication for more than 2 years (26, 28). Different definitions of asthma remission have been suggested. Studies on different populations and different evaluation periods usually yield different remission rates (29). Cai et al. (28) studied 110 children with asthma, who did no undergo the Global Initiative for Asthma (GINA) treatment for different reasons, and they found a natural remission rate (2 years without exacerbations) of 29.18%; that is, the majority of the children (70.82%) did not achieve natural remission without treatment. They also found environmental exposure to be closely associated with the natural prognosis (28).

Patients with clinical remission (absence of respiratory symptoms without anti-asthma medication) may still have bronchial hyperresponsiveness and decreased lung functions (27). Panhuysen et al. (30) studied 181 asthma patients between 13 and 44 years of age, and they found that at their 25 year follow-up surveys only 11% of them had asthma resolved (asymptomatic and free of bronchial hyperresponsiveness) although 41% of them did not report symptoms. A study conducted by researchers from the Netherlands with 30 year follow-ups on 91 individuals with asthma (5–14 years at the time of diagnosis) found that patients with lower pulmonary function during childhood had smaller lung function growths (27). Higher asthma remission rates in adulthood are usually associated with better lung function and mild symptoms in childhood. Of the 91 research subjects, 22% had complete remission and 52% (22 + 30%) had clinical remission (27). The complete remission of asthma was defined as the absence of wheezing or asthma attack for 3 years, in the absence of ICS use, and normal lung function (predicted FEV1 value >90%), and lack of bronchial hyperresponsiveness (BHR) (PC 10.16 mg/ml). Clinical remission was defined as the absence of wheezing or asthma attacks without ICS use.

Childhood asthma can affect lung development and increase the risk for developing chronic obstructive pulmonary disease (COPD) in adulthood (31). Many preschool-aged children develop wheezing symptoms after viral infections (wheezy bronchitis/viral associated wheeze [WB/VAW]). Epidemiological investigations shown that WB/VAW in childhood is closely associated with COPD due to deterioration of the ventilation function early in life, causing potential airway abnormalities that may accelerate the decline of the ventilation function during adulthood. Studies have shown that preschooler wheezing is associated with school-age/adolescent asthma and poor lung functions (32, 33). Lack of standardized treatment for childhood asthma prevents maximization of lung growth, which becomes an important risk factor for COPD (31). Patients with asthma develop lower respiratory tract ventilation dysfunction due to lower respiratory tract mucosal congestion, edema, increased exudation, and mucus plugs formation. Recurrent asthma can lead to increased air pressure in the bronchial lumen, causing long term alveolar inflation and decreased elasticity, eventually leading to COPD (20). In a cross-sectional study in Wellington, 749 adults aged 25–75 years were randomly chosen from 3,500 people to conduct a survey on medical history of childhood asthma (31). Lung function and Computed Tomography (CT) were examined and diagnosed according to the COPD golden standard, and the results showed that adults diagnosed with asthma during childhood had a 5 fold increased risk of COPD during adulthood than those without childhood asthma (31).

### The WeChat Platform and WeChat Official Accounts for Follow-Ups Improve Children's Asthma Compliance During the COVID-19 Pandemic

Compliance with physicians' instructions has been a continuing concern of pediatricians for children with asthma, because the level of adherence to treatment is directly related to the level of childhood asthma control (34). Some older children neglect use of their anti-asthma medication in the absence of parental supervision. Children's asthma compliance is closely related to parents' understanding of the condition, belief, and behavior. A study has shown that standardized health educational management for asthma control can improve parents' awareness and knowledge of the disease, and that this enhanced knowledge results in treatment adherence and behavioral changes to follow medical directions (35). With the ongoing COVID-19 pandemic, parents often postpone their follow-up visits to the hospitals in fear of the viral exposure. The WeChat platform provides a good way to quickly send texts, pictures, and videos without limitation of time or space, and is widely used. Physicians using WeChat official public accounts and the WeChat platform to establish connections with patients' family members can provide appropriate medical services, and we can promote patients' compliance. Publishing professional asthma prevention and treatment education through WeChat public accounts, physicians can strengthen the family members' awareness of asthma, instruct them to arrange long-term follow-ups, and encourage them to adhere to medication directions. At the same time, physicians can check whether the patients have mastered the inhalation technique (checking whether the mouth is tight, whether the inhalation is deep enough, and whether the time holding the breath is sufficient), and can guide children to use drugs rationally, and to correct issues in a timely manner until the correct technique is mastered. Moreover, the platform allows physicians to answer parents' and children's questions and provide guidance accordingly, to improve parents' and children's confidence in dealing with asthma, and to establish connections between patients to exchange experiences from one another for controlling the disease. Efforts have been made in multiple different directions for improving asthma control rates.

## Protection of Children With Asthma During the Pandemic

### Personal Protection From COVID-19 (36), (37)

(1) Reduce outings: physically isolating children from contact with infected individuals by staying home can help prevent infections in children, avoid public transportation if you have to go out. (2) Do not share towels, wash/dry clothes and beddings frequently. (3) Maintain a healthy work and rest schedule. (4) Keep your home ventilated, daily clean furniture surfaces, door knobs, and others. (5) Cover the toilet when flushing. (6) Keep children away from animals other than family pets, especially wild animals. (7) Follow the seven-step hand-washing method to wash your hands frequently, eat cooked food, drink plenty of water, and eat more fresh vegetables and fruits. (8) Avoid going to crowded spaces with poor ventilation, wash hands immediately after returning home. Do not touch your mouth, nose, and eyes before washing your hands. (9) Keep a good supply of common materials at home for epidemic control and prevention: thermometers, masks, household disinfection items, etc. Pay close attention to appearance of symptoms such as fever and cough. Children belong to a low-risk exposure group for SARS-CoV2. Wearing disposable medical masks or equivalent products is recommended when going to crowded places such as supermarkets or shopping malls, when taking public transportation, visiting medical institutions, and riding elevators, etc. When staying at home or at well-ventilated low-density places, masks can be removed or replaced with non-medical masks. People belonging to low-risk groups can re-use masks, and masks that need to be reused should be placed in a clean, breathable paper bag. Dirty, deformed, damaged, and smelly masks need to be replaced at once.

### Stress Reduction, Creation of a Warm Family Environment

Negative emotions such as anxiety and depression are common in children with asthma, especially among older children (3). Some parents also exhibit excessive anxiety during this pandemic period and unknowingly affect the mood of other family members. A negative family atmosphere is not healthy for children and impairs disease control (38). Negative emotions such as stress can increase the release of histamine or other allergic inflammatory mediators in the body, which inhibit reactivity of sympathetic nerves, stimulate excitability of the vagus nerve, and induce or exacerbate asthma attacks (39). A long-term follow-up study in Canada with 4,025 mothers and their children (3–5 days after child birth, at 6 months, 5 years, and 14 years) found that 14 year-old children with asthma (*n* = 1,457) had more clinical visits (4.1 vs. 2.2) than those without asthma (*n* = 2,568), that mothers of children with low DSSI scores were more likely to reach anxiety and depression thresholds (15 vs. 12%), and that the psychological pressure of mothers was associated with the frequencies of children's clinical visits for asthma (accounting for 25% of the variations in adolescent clinical visits) (40). On the other hand, applying scientific methods to educate children to maintain good living habits and reasonable recreational activities are also conducive to maintaining their inner happiness and helping them comply with medical treatments (41). In China, mothers conduct most care giving tasks, but fathers should be encouraged to participate more in childcare. During this epidemic, parents should strive to create a warm, united, and relaxed family atmosphere. Parents should educate themselves more about the epidemic and study the information released by authoritative institutions to avoid unnecessary panic. Parents can then help their children to become psychologically stronger and to organize regular life schedules, they can coach the children to alleviate negative emotions, and guide them to follow reasonable disease prevention procedures and treatment plans.

### Healthy Balanced Diet Accompanied by Regular Exercise

Extra body weight increases the risk of developing asthma, obesity is closely associated with asthma (42–44). A South Korean study on 667 children with asthma (mean age 9.47 ± 4.23 years, mean body mass index 18.6 ± 3.6 Kg/m^2^) found that asthma severity increases from moderate to severe as the body mass index changes from 18.4 ± 3.6 to 19.1 ± 3.5 Kg/m^2^ (44). When isolated at home, the activity levels are greatly reduced. A healthy balanced diet is essential for proper weight management. Avoid food allergies by reducing the intake of irritating foods. Eliminate or reduce acute asthma attack by avoiding asthma triggers.

Appropriate exercise helps to maintain a healthy weight, improves children's lung functions, improves quality of life, enhances athletic abilities, and promotes children's healthy growth and development (42). Many studies have suggested that low and medium-intensity aerobic exercise can be used as the main form of rehabilitation exercise for children with non-acute asthma (17).

When choosing a home workout space, pay attention to surrounding furniture, avoid collisions caused by excessive movements, and avoid allergen sources; do. not exercise in cold and dry environments, always warm up before the exercise routine. The recommended sports for parents to help their children include radio calisthenics, dance, and moderate household chores. You can also play musical instruments to exercise lung function or perform interesting exercises to promote enthusiasm; avoid excessive ventilation actions such as laughter and shouting during exercise; change clothing after exercising.

### Avoid Asthma Triggers (Dust Mites and Tobacco Smoke)

In North America, adults spend approximately 87% of their time in buildings (45). Adults may spend even more of their time indoors during the COVID-19 pandemic, and children are likely to stay home exclusively. Indoor air allergens affecting children with asthma can originate from outside or from the indoor environment itself. Dust mites, animal fur, cockroaches, mice, and molds are sources of indoor allergens. The other main category includes air pollutants such as particulate matter (PM), nitrogen oxides, tobacco smoke, and ozone in the air (46). Dust mites are the most common allergen causing respiratory allergic diseases, they lead to 80% of infantile asthma attacks (45), and they are the most important and persistent risk factor in the development of childhood asthma. The severity of dust mite allergic asthma symptoms is closely related to the degree of exposure to dust mites (45). Viable methods for removing dust mites in your living space include: (1) Attentive regular indoor dust removal, thorough removal of dust 1–2 times a week, and clean-up of dead corners such as those under beds. A high-powered vacuum cleaner with a dust mite removal function is recommended. (2) Regular sun drying of the beddings, sun light and ultraviolet radiation have proven simple, convenient, and safe dust mite killing effects. (3) Frequent bedding washings: change beddings every 2 weeks. (4) High temperature or frozen temperature methods remove dust mites: allergens can be removed from heat-resistant clothing after treatment at 60°C for half an hour. (5) Air-conditioning filter cleaning: The AC air supply distributes and increases indoor dust mite antigens, and AC filters should be cleaned once a month, according to the dust mites' life cycle (47).

Tobacco smoke is an important source of indoor pollution. Cigarettes and smoke contain more than 1,200 harmful components. The more important ones include soot particles, carbon dioxide, carbon monoxide, and nicotine (20). In addition to stimulant effects, tobacco smoke can activate immune cells to induce TH_2_-dominant responses, which are a trigger for asthma. Moreover, tobacco is an important cause of refractory asthma (20, 48). Tobacco can damage airway epithelial cells, cause parasympathetic hyperfunction, bronchial smooth muscle contraction, hyperthyroid gland secretion, acceleration of the lung function deterioration, and reduction of response to inhaled glucocorticoids, and may cause uncontrollable asthma (49). Passive smoking reduces the likelihood of asthma remission in children, and this effect persists until mid-age (26).

### Early Diagnosis, Treatment, and Management of Acute Asthma Attacks

Early symptoms of an acute asthma attack may include any of the following: difficulty breathing, increased wheezing, shortness of breath, increased coughing (especially night coughing), fatigue or reduced exercise tolerance, difficulty feeding, poor response to relieving drugs, chest tightness, belching, and mental stress (50). When an acute attack of asthma occurs, its severity needs to be assessed. Mild attacks generally do not affect children's activities, they can speak normally, they can lie flat, and present no cyanosis (17). Pre-interventions can usually be performed for children suffering mild asthma attacks. The concept of pre-intervention is based on use of intensive control drugs for short-term treatment of patients with acute respiratory infections or signs of asthma attacks, instead of continuous use of long-term control drugs. The biggest advantage of the pre-intervention scheme is that it reduces the overall drug load, while achieving good asthma control (11). For acute onset of mild to moderate asthma, high-dose budesonide suspension (1 mg/time) is used in combination with inhaled short-acting β-2 RA aerosol as the initial treatment, 2 times a day, or as necessary (repeat administrations once every 4–6 h). Then the interval between doses can be extended as appropriate, depending on the recovery conditions, and sustaining treatment for 7–10 days (21). Immediate hospitalization is required when one of the following symptoms are observed (50) (1) no-response after inhalation of short-acting β-2 RA 3 times within 1–2 h; (2) shortness of breath after three administrations of short-acting β-2 receptor, (normal breathing frequency is <60 times/min for infants from 0–2 months; <50 times/min for infants from 2–12 months; and <40 times min for children from 1–5 years); (3) patient is unable speak or drink due to shortness of breath; (4) presence of cyanosis; (5) intercostal depression observed; (6) inhaled air oxygen saturation lower than 92%.

## Conclusion

The prevalence of asthma has increased in China. Non-standardized treatments and poor medical compliance have led to less-than-ideal total control rates. Awareness of the disease is important to ensure proper interventions during the critical period of children's growth and development. In addition to preventing and controlling SARS-CoV2 infections during the pandemic, standardized asthma treatments should be continued and adhered to for improving outcomes of children with asthma.

## Data Availability Statement

The original contributions presented in the study are included in the article/supplementary material, further inquiries can be directed to the corresponding author.

## Author Contributions

BD conceived and designed the study. BD and YL wrote the draft of this manuscript. YL edited the manuscript. Both authors contributed to the article and approved the submitted version.

## Conflict of Interest

The authors declare that the research was conducted in the absence of any commercial or financial relationships that could be construed as a potential conflict of interest.
